# Efficacy, safety, and survival of neoadjuvant immunochemotherapy in operable non-small cell lung cancer: a systematic review and meta-analysis

**DOI:** 10.3389/fimmu.2023.1273220

**Published:** 2023-12-01

**Authors:** Yue Zheng, Baijie Feng, Jingyao Chen, Liting You

**Affiliations:** ^1^ West China School of Medicine, Sichuan University, Chengdu, Sichuan, China; ^2^ Precision Medicine Research Center, West China Hospital, Sichuan University, Chengdu, Sichuan, China; ^3^ Department of Laboratory Medicine, West China Hospital, Sichuan University, TCM Regulating Metabolic Diseases Key Laboratory of Sichuan Province, Hospital of Chengdu University of Traditional Chinese Medicine, Chengdu, Sichuan, China

**Keywords:** non-small cell lung cancer, neoadjuvant immunochemotherapy, efficacy, safety, survival

## Abstract

**Background:**

Neoadjuvant immunochemotherapy may benefit patients with non-small cell lung cancer (NSCLC), but its impact requires further investigation.

**Methods:**

A meta-analysis was conducted. PubMed, Embase, Web of Science, and the Cochrane Library were searched. The study was registered in PROSPERO (registration no. CRD42022360893).

**Results:**

60 studies of 3,632 patients were included. Comparing with neoadjuvant chemotherapy, neoadjuvant immunochemotherapy showed higher pCR (RR: 4.71, 95% CI: 3.69, 6.02), MPR (RR, 3.20, 95% CI: 2.75, 3.74), and ORR (RR, 1.46, 95% CI: 1.21, 1.77), fewer surgical complications (RR: 0.67, 95%CI: 0.48, 0.94), higher R0 resection rate (RR: 1.06, 95%CI: 1.03, 1.10, I^2^ = 52%), and longer 1-year and 2-year OS, without affecting TRAEs. For neoadjuvant immunochemotherapy in NSCLC, the pooled pCR rate was 0.35 (95% CI: 0.31, 0.39), MPR was 0.59 (95% CI: 0.54, 0.63), and ORR was 0.71 (95% CI: 0.66, 0.76). The pooled incidence of all grade TRAEs was 0.70 (95% CI: 0.60, 0.81), and that of >= grade 3 TRAEs was 0.24 (95% CI: 0.16, 0.32). The surgical complications rate was 0.13 (95% CI: 0.07, 0.18) and R0 resection rate was 0.98 (95% CI: 0.96, 0.99). The pooled 1-year OS was 0.97 (95%CI: 0.96, 0.99), and 2-year OS was 0.89 (95%CI: 0.83, 0.94). Patients with squamous cell carcinoma, stage III or higher PD-L1 performed better. Notably, no significant differences were observed in pCR, MPR, and ORR between 2 or more treatment cycles. Pembrolizumab-, or toripalimab-based neoadjuvant immunochemotherapy demonstrated superior efficacy and tolerable toxicity.

**Conclusion:**

According to our analysis, reliable efficacy, safety, and survival of neoadjuvant immunochemotherapy for operable NSCLC were demonstrated.

**Systematic review registration:**

https://www.crd.york.ac.uk/prospero/display_record.php?ID=CRD42022360893, identifier CRD42022360893.

## 1 Introduction

Non-small cell lung cancer (NSCLC) remains the main reason of tumor-related deaths (1). Of patients with NSCLC, 20-25% are surgically resectable, but 30-55% of patients treated with surgery still experience cancer relapse, metastasis, or death (2, 3). Due to the large tumor burden in advanced NSCLC, direct surgical treatment is challenging, while neoadjuvant therapy can shrink the tumor and make unresectable tumors resectable (4, 5). As a result, the NCCN guidelines recommend preoperative neoadjuvant therapy and postoperative adjuvant therapy as the standard therapy for NSCLC (6). But neoadjuvant chemotherapy may only provide a 5-6% benefit of 5-year overall survival (OS) and few patients achieve pathologic complete response (pCR) (7).

Immune checkpoint inhibitor (ICI) plays an important role in the first-line and second-line therapy of patients with NSCLC, showing a better survival benefit than chemotherapy (8–12). A growing view is that earlier immunotherapy may provide greater benefits. Several clinical studies have shown that neoadjuvant immunotherapy can was crucial in the comprehensive treatment of NSCLC, with controllable adverse events and less surgical delay (13, 14). CheckMate 159 showed that the pCR and MPR rates of nivolumab were 10% and 45%, respectively (15). The LCCMC 3 study revealed that the MPR rate of patients receiving 2 cycles of atezolizumab neoadjuvant therapy was 20.4%, and the pCR rate was 6.8% (16). These results were superior to those of previous neoadjuvant chemotherapy.

In the NADIM study, neoadjuvant immunochemotherapy for operable NSCLC had a pCR rate of 69.2% and an MPR rate of 84.6% (17, 18). In the NeoTPD01, and NCT02716038, and SAKK 16/14 studies, the MPR was around 60% (19–21). CheckMate 816, the first successful phase III trial of neoadjuvant immunochemotherapy versus chemotherapy in operable stage IB-III NSCLC, showed that MPR (36.9% *vs*. 8.9%) and pCR (24% *vs*. 2.2%) were significantly improved (22, 23). Updated data from the NADIM II study also revealed that neoadjuvant immunochemotherapy can effectively shrink tumors, increase pCR (36.8% *vs*. 6.9%), MPR (52.6% *vs*. 13.8%), and ORR (75.4% *vs*. 48.2%), and help patients obtain better survival in locally advanced IIIA-IIIB resectable NSCLC (24, 25). These studies provide encouraging results of neoadjuvant immunochemotherapy in patients with NSCLC.

Despite the promising results, concerns about the efficacy, safety, and survival of neoadjuvant immunochemotherapy remain. To address these concerns, we conducted a meta-analysis to combine all related trials and evaluate the efficacy, safety, and survival rates of neoadjuvant immunochemotherapy in operable NSCLC. Additionally, we compared the results among different subgroups, such as age, gender, smoking history, histology, stage, treatment cycles, pretreatment programmed death-ligand 1 (PD-L1), and ICI type. Our goal was to provide guidance for the clinical treatment of NSCLC.

## 2 Methods

To ensure the accuracy and transparency of our study, we followed the PRISMA (Preferred Reporting Items for Systematic Reviews and Meta-Analyses) and AMSTAR (Assessing the methodological quality of systematic reviews) guidelines (26, 27). This study was registered in PROSPERO (registration no.CRD42022360893, available at: https://www.crd.york.ac.uk/prospero/display_record.php?ID=CRD42022360893).

### 2.1 Data search

We searched PubMed, Embase, Web of Science, and Cochrane Library, using keywords such as “neoadjuvant”, “immunotherapy”, “chemotherapy”, and “non-small cell lung cancer”. The search was conducted independently by two authors and included papers updated until July 2023. Language restrictions were not applied.

### 2.2 Study criteria

To be eligible for our study, trials were required to have administered neoadjuvant immunochemotherapy to patients diagnosed with operable NSCLC and acquired radiological or pathological response data. Enrolled patients should not have received any prior systemic anti-neoplastic treatment for NSCLC, must have had no history of lung radiotherapy, and should have undergone surgical resection for NSCLC. Excluded were trials involving patients with concurrent progressive or actively treated additional malignancies, those who had received previous systemic antineoplastic therapy for NSCLC, or those with a history of lung radiotherapy. Studies falling into categories such as reviews, comments, case reports, trial protocols, or animal experiments were also eliminated. In the context of randomized controlled trials (RCTs), preference was given to those that compared non-combination therapy with combination therapy, establishing a basis for a comparison group. In cases where multiple publications reported results from the same study population across different journals, the most comprehensive or most current study was selected for inclusion.

### 2.3 Data extraction and quality assessment

We screened these literatures based on pre-determined inclusion and exclusion criteria. Two authors independently screened the records, read the full-text papers, and extracted details from the included studies. The primary endpoints were pCR (no residual vital cancer cells), major pathologic response (MPR, <= 10% residual vital cancer cells), the incidence of grade 3 or higher treatment-related adverse events (TRAEs), and 1-year and 2-year OS (the duration from randomization to death from any reason). The secondary endpoints were objective response rate (ORR, proportion of patients with a partial or complete response), total grade TRAEs, R0 resection rate, and the incidence of surgical complications. We assessed the quality of RCTs using the Cochrane Collaboration’s tool (28). For dual-arm non-RCTs, we used the Newcastle-Ottawa scale, and for single-arm non-RCTs, we used the Methodological Index for Non-Randomized Studies criteria (MINORS) to assess quality (29, 30). We consulted a third referee to resolve discrepancies.

### 2.4 Data synthesis and statistical analysis

For single-arm studies, we combined the proportion of each endpoint with a 95% confidence interval (95% CI) to draw forest plots. For dual-arm studies, we calculated the risk ratio (RR) and 95% CI. We used the Cochrane Q test and I^2^ test to determine statistical heterogeneity. If I^2^ > 50% or p < 0.05, we used the random effects model. If I^2^ < 50% or p > 0.05, we used the fixed effects model. We carried out subgroup analysis according to age, gender, smoking history, histology, stage, treatment cycles, pretreatment PD-L1, and ICI type. The sensitivity analysis evaluated the stability of the results by ruling out each trial separately. We evaluated publication bias using funnel plots. We used R 4.3.1 software and the meta_v6.2-1 package for the analysis.

## 3 Results

### 3.1 Study selection

**Figure 1:**
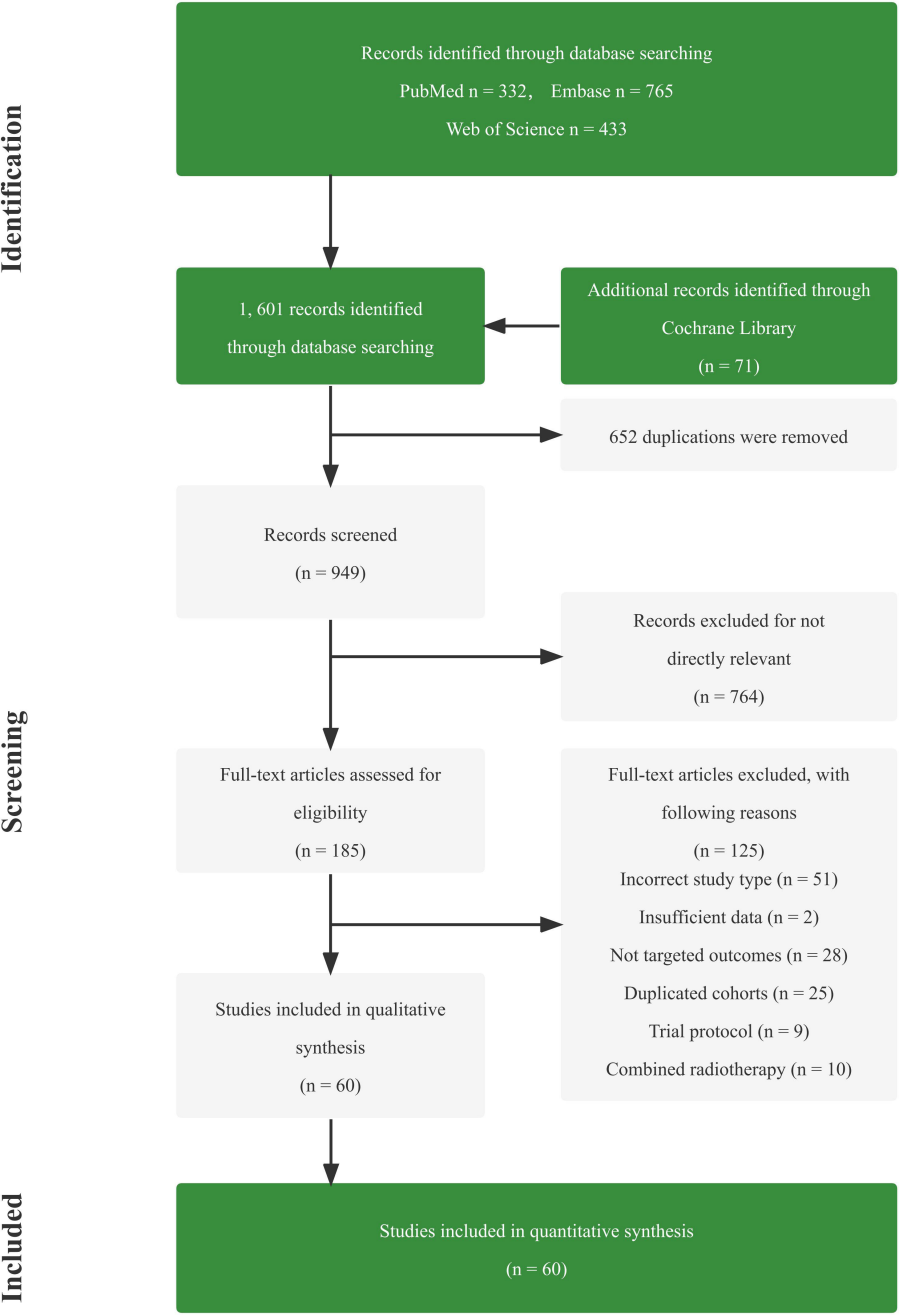
PRISMA diagram of identifying eligible studies.

Totally, 1,601 studies were screened. After eliminating duplicates and irrelevant studies based on their titles and abstracts, 1,416 were excluded, and the remaining 185 studies were reviewed in detail. Out of these, 125 studies were further excluded due to reasons such as incorrect study type, insufficient data, non-targeted outcomes, duplicated cohorts, trial protocol, and treatment combined with radiotherapy. Ultimately, 60 studies comprising 4 RCTs, 13 dual-arm cohorts, and 43 single-arm studies were selected for analysis, with a total of 3,632 eligible patients included. **Figure 1**; **Supplementary Table 1** provides details of this literature search. **Tables 1**, **2** suggest the characteristics of the eligible studies. All the included studies were considered moderately or highly credible, and **Supplementary Figure 3** provides funnel plots. The quality scores of each eligible study are presented in **Supplementary Tables 2**-**4**.

**Table 1:** Main characteristics of dual-arm studies included in the meta-analysis.

Author (Year)	Study (Phase, Design)	Registered ID (Randomization)	Study type	Sample size	Stage	Median age	Gender (M/F)	Smoking history (%)	Median follow-up (month)	Neoadjuvant therapy of intervention Arm	Neoadjuvant therapy of control Arm	Surgery	Outcomes
Provencio (2022) (24, 25)	NADIM II (II, open-label)	NCT03838159 (1:1)	RCT	86 (57/29)	IIIA-IIIB	_	_	_	_	nivolumab + paclitaxel + carboplatin	paclitaxel + carboplatin	53/20	pCR, MPR, ORR, TRAE
Forde (2023) (23)	CheckMate 816 (III, open-label)	NCT02998528 (1:1)	RCT	358 (179/179)	IB-IIIA	64.5	255/103	88.8	41.4	nivolumab + cisplatin or carboplatin	cisplatin or carboplatin	149/135	pCR, MPR, ORR, TRAE, OS, R0 resection rate
Wakelee (2023) (31)	KEYNOTE-671 (III, double-blind)	NCT03425643 (1:1)	RCT	797 (397/400)	II-III	63.5	563/234	87.3	25.2	pembrolizumab + chemo	cisplatin-based chemo	325/317	pCR, MPR, TRAE
Alì (2023) (32)	_	(12:14)	Retro	26 (12/14)	I-IV	69	18/8	_	29	ICI + chemo	platinum based doublet chemo	26	pCR, MPR
Cesur (2022) (33)	_	_	Retro	65 (17/48)	_	_	_	_	_	ICI + chemo	chemo	17/48	MPR, OS
Sun (2022) (34)	_	_	Retro	81(40/41)	II-IIIA	_	57/24	77.8	24	ICI + chemo	chemo	40/41	pCR, MPR
Dai (2022) (35)	_	ChiCTR2200060433(20:42)	Retro	62 (20/42)	IB-IIIB	_	53/9	74.2	24	ICI + chemo	chemo	20/42	pCR, MPR, R0 resection rate
Sun (2022) (36)	_	_	Retro	168 (79/89)	II-IIIA	61	136/32	92.9	18	ICI + chemo	chemo	79/89	pCR, MPR
Feng (2022) (37)	_	(8:13)	Pro	21 (8/13)	IIA-IIIB	64	20/1	95.2	20.53	pembrolizumab or toripalimabd + gemcitabine, paclitaxel or nab-paclitaxel + cisplatin or carboplatin	gemcitabine, paclitaxel or nab-paclitaxel + cisplatin or carboplatin	8/13	pCR, MPR, ORR, TRAE, R0 resection rate
Hou (2022) (38)	_	(31:25)	Pro	56 (31/25)	IIIA-IIIB	60.7	43/13	82.1	11.8	camrelizumab + paclitaxel + carboplatin	paclitaxel + carboplatin	31/25	pCR, MPR, ORR, TRAE, OS
Liu (2022) (39)	_	(79:91)	Retro	170 (79/91)	IB–IIIB	_	141/29	82.9	17.0	pembrolizumab or nivolumab or sintilimab or camrelizumab + paclitaxel or pemetrexed or docetaxel or gemcitabine + platinum	paclitaxel or pemetrexed or docetaxel or gemcitabine + platinum	79/91	pCR, MPR, ORR, R0 resection rate
Yue (2022) (40)	_	(1:1)	Retro	18 (12/6)	I-III	62.5	17/5	81.8	17.7	ICI + chemo	chemo	12/6	pCR, MPR
Zhang (2022) (41)	_	(1:2)	Retro	190 (69/121)	IB-IIIB	_	175/15	85.3	ICI + chemo: 18.6 chemo: 22.4	PD-1 + cisplatin or carboplatin	cisplatin or carboplatin	69/121	pCR, MPR, ORR, TRAE, OS, R0 resection rate, surgical complications
Zhao (2022) (42)	_	(42:98)	Retro	140 (42/98)	IB-IIIB	_	123/17	50	ICI + chemo: 18 chemo: 24	ICI + chemo	chemo	42/98	pCR, MPR, ORR, R0 resection rate, surgical complications
Liang (2021) (43)	_	(1:1)	Retro	20 (10/10)	IIB-IIIB	60.89	14/6	70	ICI + chemo: 13.5 chemo: 20.8	pembrolizumab or nivolumab or sintilimab + platinum-based doublet chemo	platinum-based doublet chemo	10/10	pCR, MPR, ORR, OS, surgical complications
Sun (2021) (44)	_	(1:1)	Retro	168 (79/89)	II-IIIA	_	136/32	79.7		nivolumab or camrelizumab or tislelizumab + chemo	chemo	79/89	pCR, MPR
Lei (2020) (45)	(II, open-label)	NCT04338620 (1:1)	RCT	27 (14/13)	IIIA-IIIB	_	_	_	_	camrelizumab + ab-Pac + cisplatin	ab-Pac + cisplatin	13 (7/6)	pCR, MPR, ORR, TRAE

RCT, randomized controlled trials; pCR, pathologic complete response; MPR, major pathologic response; ORR, objective response rate; TRAE, treatment related adverse events; EFS, event free survival; OS, overall survival; Pro, prospective study; DFS, disease free survival; Retro, retrospective study; ICI, immune checkpoint inhibitors; chemo, chemotherapy; PD-1, programmed death 1; ab-Pac, albumin-bound paclitaxel.

**Table 2:** Main characteristics of single-arm studies included in the meta-analysis.

Author (Year)	Study (Phase, Design)	Registered ID	Study type	Sample size	Stage	Median age (Years)	Gender (M/F)	Smoking history (%)	Median follow-up (month)	Neoadjuvant therapy of intervention Arm	Surgery	Outcomes
Henick (2023) (46)	(I, open-label)	_	Pro	30	_	_	_	_	39.5	atezolizumab +carboplatin+ nab-paclitaxel	29	pCR, MPR, OS, R0 resection rate
Tao (2023) (47)	(II, open-label)	NCT04606303	Pro	55	IIB-IIIB	62	50/5	89.1	_	toripalimab + chemo	48	pCR, MPR, TRAE, R0 resection rate
Fang (2023) (48)	_	_	Retro	211	IB–IIIB	64	196/15	85.8	17	ICI + chemo	211	pCR, MPR, ORR, TRAE, R0 resection rate, surgical complications
Cascone (2023) (49)	NEOSTAR (II, open-label)	NCT03158129	Pro	22	IB–IIIA	69.5	10/12	77.3	39.2	nivolumab + chemo	22	pCR, MPR, ORR, TRAE, R0 resection rate
Chen (2023) (50)	_	_	Retro	61	_	_	_	_	_	pembrolizumab + chemo	_	pCR, MPR
Zhao (2023) (51)	_	_	Retro	25	IIB-IIIB	65	22/3	_	_	pembrolizumab + carboplatin or cisplatin+ pemetrexed or nab-paclitaxel	21	pCR, MPR
Han (2023) (52)	_	_	Retro	29	III-IV	_	21/8	72.41	_	ICI + chemo	29	pCR, MPR, ORR, R0 resection rate
Hu (2023) (53)	_	_	Retro	101	IIB-IIIC	58	93/8	64.4	12	ICI + chemo	101	pCR, MPR, ORR, OS, R0 resection rate
Zhuang (2023) (54)	_	_	Retro	129	IIA-IIIB	63	117/12	72.1	_	ICI + chemo	129	pCR, MPR, ORR
Wu (2023) (55)	(Ib/III, double-blind)	NCT04316364	Pro	37	I-III	_	_	_	_	adebrelimab (SHR-1316) + nab-paclitaxel + carboplatin	34	pCR, MPR, ORR, TRAE, surgical complications
Wang (2022) (56)	(II, open-label)	NCT04865705	Pro	33	IIIA-IIIB	_	_	_	_	ICI + chemo	18	pCR, MPR, DFS, OS, R0 resection rate
Zhang (2022) (57)	(II, open-label)	ChiCTR2100044645	Pro	26	IIB-IIIB	_	_	_	_	camrelizumab + albumin paclitaxel + carboplatin or cisplatin	17	pCR, MPR, TRAE, surgical complications
Xu (2022) (58)	_	_	Retro	14	IIIA-IIIB	68	14/0	85.71	_	ICI + chemo	11	pCR, MPR, ORR, TRAE, R0 resection rate, surgical complications
Lin (2022) (59)	(II, open-label)	NCT05244837	Pro	37	IIB-III	63	31/6	_	_	tislelizumab + carboplatin + pemetrexed or nab-paclitaxel	27	pCR, MPR, TRAE, R0 resection rate
Dong (2022) (60)	(II, open-label)	NCT04897386	Pro	14	III	64.5	_	_	9.5	durvalumab + chemo	10	pCR, MPR, TRAE, OS, R0 resection rate
Ma (2022) (61)	_	_	Retro	59	IIA-IIIB	61.34	50/9	72.9	_	ICI + chemo	59	pCR, R0 resection rate
Dai (2022) (62)	_	ChiCTR1900023758, NCT04379739, and off-trial	Retro	23	IIB, IIIA-B	63.2	22/1	60.9	15	ICI + chemo	23	pCR, MPR, ORR, TRAE, R0 resection rate, surgical complications
Faehling (2022) (63)	KOMPASSneoOP	_	Retro	59	IIB-IVB(44%)	63.6	30/29	95	24.3	ICI + chemo	59	pCR, MPR, ORR, PFS, OS
Sun (2022) (64)	(II, open-label)	NCT04326153	Pro	20	IIIA-B	59.5	18/2	90	_	sintilimab + nab-paclitaxel + carboplatin	16	pCR, MPR, ORR, DFS, OS, TRAE, R0 resection rate
Gao (2022) (65)	(open-label)	ChiCTR2200057840	Pro	44	IIIA-B	61.5	33/11	33 (75.0)	_	ICI + chemo	44	pCR, MPR, TRAE, R0 resection rate, surgical complications
Qiu (2022) (66)	neoSCORE (II, open-label)	NCT04459611	Pro	60	IB-IIIA	_	_	_	_	sintilimab + carboplatin + nab-paclitaxel or pemetrexed	29	pCR, MPR, ORR, TRAE, DFS, OS
Wu (2022) (67)	_	_	Pro	76	IB-IIIB	62	72/4	67	12.2	pembrolizumab or nivolumab + chemo	76	pCR, MPR, ORR, TRAE, R0 resection rate
Zhai (2022) (68)	_	_	Retro	46	IIIA-IIIB	63	26/20	93.5	15.5	nivolumab + paclitaxel + carboplatin	45	pCR, MPR, TRAE, DFS, OS, R0 resection rate, surgical complications
Zhang (2022) (69)	(II)	ChiCTR1900023758	Pro	50	IIIA	64.84	44/6	76	13.6	sintilimab + carboplatin, gemcitabine or pemetrexed	30	pCR, MPR, ORR, TRAE, DFS, OS, R0 resection rate, surgical complications
Yan (2021) (70)	Renaissance Study (II, open-label)	NCT04606303	Pro	21	IIB-IIIB	62	19/2	85.7	_	toripalimab + cisplatin-based chemo	19	pCR, MPR, TRAE, R0 resection rate, surgical complications
Rothschild (2021) (21)	SAKK 16/14 (II, open-label)	NCT02572843	Pro	67	IIIA(N2)	61	35/32	95.5	28.6	cisplatin + docetaxel + durvalumab	55	pCR, MPR, ORR, TRAE, EFS, OS, R0 resection rate
Chen (2021) (71)	_	_	Retro	12	IIIA-IIIB	61	9/3	75	18.17	pembrolizumab or nivolumab + carboplatin + paclitaxel	12	pCR, MPR, TRAE, surgical complications
Chen (2021) (72)	_	_	Retro	35	IIIA-IIIB	_	29/6	77.1	13.29	pembrolizumab + cisplatin + paclitaxel or pemetrexed + paclitaxel	35	pCR, MPR, TRAE, PFS, OS, R0 resection rate
Duan (2021) (73)	(open-label)	_	Pro	23	IIA-IIIB	61.83	22/1	95.7	_	ICI + chemo	20	pCR, MPR, ORR, TRAE, PFS, R0 resection rate, surgical complications
Hong (2021) (74)	_	_	Retro	25	II–III	_	23/2	68	_	pembrolizumab or sintilimab or camrelizumab + taxol + cisplatin or carboplatin	25	pCR, MPR, ORR, R0 resection rate, surgical complications
Hu (2021) (75)	_	_	Pro	20	IB-IIIB	56	18/2	85	_	sintilimab or pembrolizumab or toripalimab + chemo	20	pCR, MPR, ORR, TRAE, R0 resection rate, surgical complications
Shi (2021) (76)	_	_	Retro	27	IIA-IIIB	_	_	_	_	camrelizumab or toripalimab or tislelizumab or sintilimab or pembrolizumab + chemo	27	pCR, MPR, TRAE
Zhang (2021) (77)	(II)	NCT04144608	Pro	18	IIIA-IIIB	57	13/2	_	6	toripalimab + nab-paclitaxel or pemetrexed + carboplatin or cisplatin	15	pCR, MPR, TRAE, R0 resection rate, surgical complications
Zhao (2021) (19, 78)	NeoTPD01 (II)	NCT04304248	Pro	33	IIIA-IIIB	61	27/6	_	4.13	toripalimab + carboplatin + pemetrexed or nab-paclitaxel	30	pCR, MPR, TRAE, EFS, R0 resection rate, surgical complications
Zhou (2021) (79)	_	_	Retro	20	IB-IIIB	_	17/3	85	_	pembrolizumab or toripalimab or sintilimab or camrelizumab + chemo	17	pCR, MPR, ORR, TRAE, R0 resection rate, surgical complications
Shen (2021) (80)	_	_	Pro	37	IIB-IIIB	62.8	35/2	83.8	7	pembrolizumab + ab-Pac + carboplatin	37	pCR, MPR, ORR, TRAE, R0 resection rate, surgical complications
Zhang (2021) (81)	_	NCT04324151	Retro	56	IIIA-IIIB	58	54/2	_	11	toripalimab or pembrolizumab + platinum-doublet chemo	45	pCR, MPR, TRAE, R0 resection rate, surgical complications
Provencio (2020) (18)	NADIM (II, open-label)	NCT03081689	Pro	46	IIIA	63	34/12	100	24	paclitaxel + carboplatin + nivolumab	41	pCR, MPR, ORR, TRAE, PFS, OS, R0 resection rate, surgical complications
Shu (2020) (20)	(II, open-label)	NCT02716038	Pro	30	IB-IIIA	67	15/15	100	12.9	atezolizumab + nab-paclitaxel + carboplatin	29	pCR, MPR, ORR, TRAE, DFS, OS, R0 resection rate, surgical complications
Tfayli (2020) (82)	_	NCT03480230	Pro	15	IB-III	65	7/8	73	10	chemo + avelumab	11	pCR, MPR, ORR, TRAE, surgical complications
Zinner (2020) (83)	_	_	Pro	13	IB-IIIA	69	8/5	_	10	nivolumab + cisplatin + pemetrexed or gemcitabine	13	pCR, MPR, TRAE
Liu (2020) (84)	_	_	Pro	13	II-III	63.4	11/2	84.6	3.1	pembrolizumab or toripalimab + platinum-doublet chemo	5	pCR, MPR, ORR, TRAE, R0 resection rate, surgical complications
Hilbe (2015) (85)	INN06 (II, open-label)	Eudract-Nr: 2006-004639-31	Pro	41	IB-IIIB	57.5	24/15	_	44.2	cisplatin + docetaxel + cetuximab	37	pCR, ORR, TRAE, PFS, OS

Retro, retrospective study; PD-1, programmed death 1; pCR, pathologic complete response; MPR, major pathologic response; ORR, objective response rate; TRAE, treatment related adverse events; chemo, chemotherapy; PFS, progression free survival; OS, overall survival; Pro, prospective study; DFS, disease free survival; EFS, event free survival; ab-Pac, albumin-bound paclitaxel.

### 3.2 Efficacy of neoadjuvant immunochemotherapy

**Figure 2:**
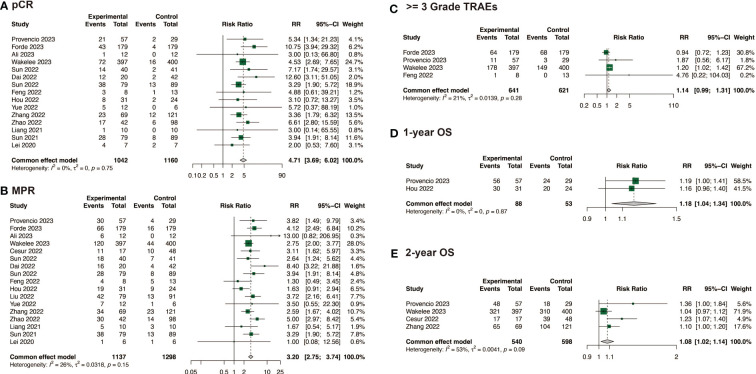
Comparison of efficacy, safety and survival between neoadjuvant immunochemotherapy with neoadjuvant chemotherapy alone. **(A)** Comparison of pCR; **(B)** Comparison of MPR; **(C)** Comparison of >= 3 Grade TRAEs; **(D)** Comparison of 1-year OS; **(E)** Comparison of 2-year OS.

**Figure 3:**
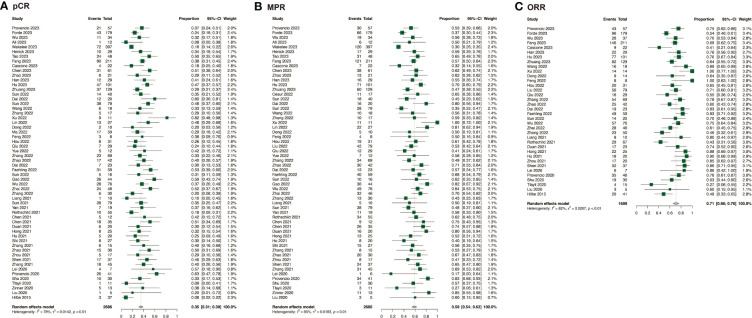
Efficacy of neoadjuvant immunochemotherapy in resectable non-small cell lung cancer. **(A)** pCR of neoadjuvant immunochemotherapy in resectable non-small cell lung cancer; **(B)** MPR of neoadjuvant immunochemotherapy in resectable non-small cell lung cancer; **(C)** ORR of neoadjuvant immunochemotherapy in resectable non-small cell lung cancer.

The efficacy of neoadjuvant immunochemotherapy was assessed based on pCR, MPR, and ORR rates, with neoadjuvant immunochemotherapy showing significantly better efficacy than neoadjuvant chemotherapy. The estimated RR was 4.71 (95% CI: 3.69, 6.02, I^2^ = 0%) for pCR, 3.20 (95% CI: 2.75, 3.74, I^2^ = 26%) for MPR, and 1.46 (95% CI: 1.21, 1.77, I^2^ = 62%) for ORR (**Figures 2A, B**; **Supplementary Figure 1A**). For neoadjuvant immunochemotherapy in NSCLC, the pooled pCR rate was 0.35 (95% CI: 0.31, 0.39, I^2^ = 78%), the MPR rate was 0.59 (95% CI: 0.54, 0.63, I^2^ = 85%), and the ORR rate was 0.71 (95% CI: 0.66, 0.76, I^2^ = 82%) (**Figure 3**).

### 3.3 Safety of neoadjuvant immunochemotherapy

**Figure 4:**
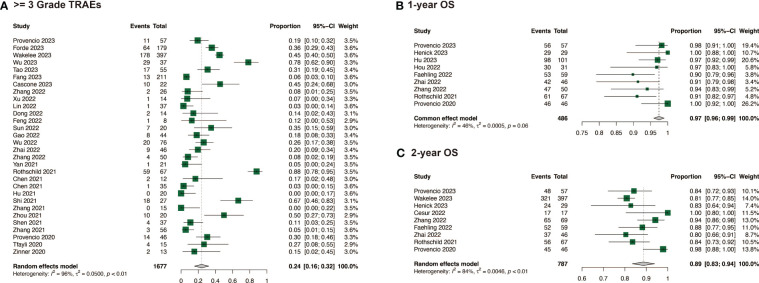
Safety and survival of neoadjuvant immunochemotherapy in resectable non-small cell lung cancer. **(A)** >= 3 Grade TRAEs of neoadjuvant immunochemotherapy in resectable non-small cell lung cancer; **(B)** 1-year OS of neoadjuvant immunochemotherapy in resectable non-small cell lung cancer; **(C)** 2-year OS of neoadjuvant immunochemotherapy in resectable non-small cell lung cancer.

In the comparison of safety and surgical outcomes between neoadjuvant immunochemotherapy and neoadjuvant chemotherapy, the estimated RR for >= grade 3 TRAEs was 1.14 (95%CI: 0.99, 1.31, I^2^ = 21%) (**Figure 2C**) and for all grade TRAEs, the RR was 1.00 (95%CI: 0.96, 1.03, I^2^ = 19%), suggesting no significant difference (**Supplementary Figure 1B**). However, neoadjuvant immunochemotherapy may result in fewer surgical complications (RR: 0.67, 95%CI: 0.48, 0.94, I^2^ = 0%) and higher R0 resection rate (RR: 1.06, 95%CI: 1.03, 1.10, I^2^ = 52%) (**Supplementary Figures 1C, D**). The pooled incidence of >= grade 3 TRAEs was 0.24 (95%CI: 0.16, 0.32, I^2^ = 96%)(**Figure 4A**). The pooled incidence of all grade TRAEs was 0.70 (95%CI: 0.60, 0.81, I^2^ = 97%) and that of surgical complications was 0.13 (95%CI: 0.07, 0.18, I^2^ = 82%), and the R0 resection rate was 0.98 (95%CI: 0.96, 0.99, I^2^ = 61%) (**Supplementary Figure 2**).

### 3.4 Survival of neoadjuvant immunochemotherapy

When compared with neoadjuvant chemotherapy, neoadjuvant immunotherapy may significantly enable long survival for patients, with a RR of 1.18 (95%CI: 1.04, 1.34, I^2 = ^0%) for 1-year OS, and 1.08 (95%CI: 1.02, 1.14, I^2^ = 53%) for 2-year OS (**Figures 2D, E**). Among the studies that reported specific survival data for patients with NSCLC receiving neoadjuvant immunochemotherapy, the pooled results were 0.97 (95%CI: 0.96, 0.99, I^2^ = 46%) for 1-year OS, and 0.89 (95%CI: 0.83, 0.94, I^2^ = 84%) for 2-year OS (**Figures 4B, C**).

### 3.5 Sensitivity analysis and subgroup analysis

**Figure 5:**
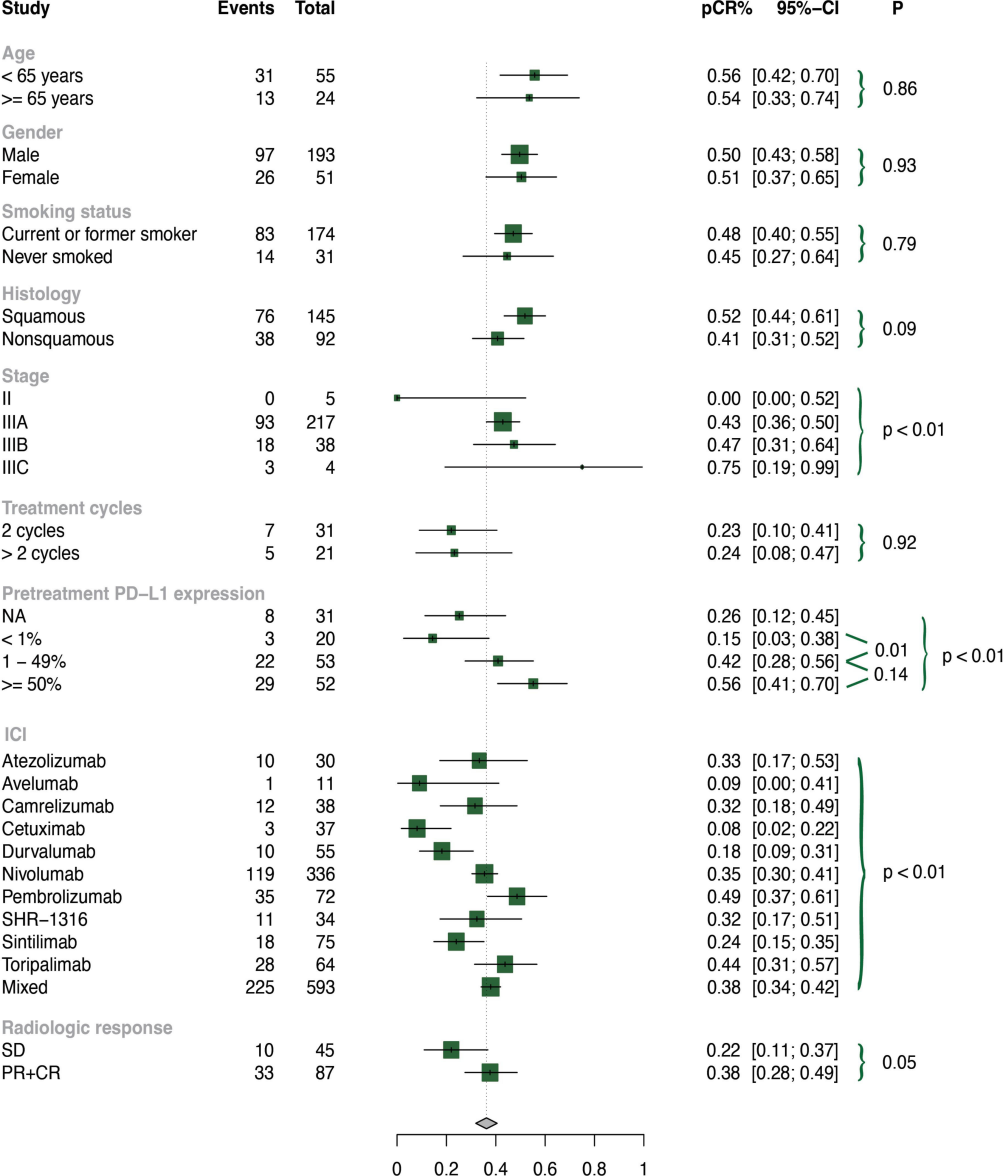
Subgroup analysis of pCR by clinical characteristics.

To test the stability, we performed sensitivity analyses by removing each individual trial, and found that our selected studies were reliable (**Supplementary Figure 4**). We also performed subgroup analyses, and the results are presented in **Figure 5**; **Supplementary Figures 5**-**7**.

As basic clinical characteristics may contribute to heterogeneity, we conducted subgroup analyses based on age, gender, and smoking history in the neoadjuvant immunochemotherapy group. However, no significant differences were found in these subgroups (all p values > 0.05).

Among the included patients, the histology subtypes were divided into squamous and non-squamous. Neoadjuvant immunochemotherapy treatment in patients with squamous lung cancer performed significantly higher rates of MPR (p = 0.03) and ORR (p < 0.01), and a tendency towards better pCR without reaching statistical significance (p = 0.09). Stage is also a key factor of heterogeneity, so we further explored subgroups based on stage (II, IIIA, IIIB, IIIC). We found that patients with stage II NSCLC experienced less benefit in terms of pCR (p < 0.01) and ORR (p = 0.05) than those with advanced stage.

The optimal treatment cycle for neoadjuvant immunochemotherapy remains uncertain, with no clear evidence indicating whether 2 or more cycles are superior. To investigate this, we conducted a subgroup analysis of treatment cycles (2 cycles *vs*. >2 cycles) and found no significant discrepancies in pCR (p = 0.92), MPR (p = 0.80), or ORR (p = 0.61) between these subgroups. We also examined the effect of pretreatment PD-L1 expression and found that patients with higher PD-L1 (TPS ≥ 50% or TPS = 1-49%) had significantly improved pCR, MPR, and ORR compared to those with lower PD-L1 (TPS < 1%). Patients who achieved partial response (PR) or complete response (CR) had higher MPR rates than those with stable disease (SD) (p < 0.01).

We observed significant differences among subgroups in pCR, MPR, ORR, and 3 or higher grade TRAEs for different ICI types (p < 0.01). Pembrolizumab-based neoadjuvant immunochemotherapy demonstrated higher pCR (0.49, 95% CI: 0.37-0.61), MPR (0.69, 95% CI: 0.57-0.80), and ORR (0.86, 95% CI: 0.71-0.95) rates. Toripalimab-based neoadjuvant immunochemotherapy showed higher pCR (0.44, 95% CI: 0.31-0.57) and MPR (0.61, 95% CI: 0.48-0.73) rates. Nivolumab-based neoadjuvant immunochemotherapy had higher pCR (0.35, 95% CI: 0.30-0.41) and ORR (0.62, 95% CI: 0.56-0.67) rates. In contrast, avelumab-based neoadjuvant immunochemotherapy demonstrated relatively lower pCR (0.09, 95% CI: 0.00-0.41), MPR (0.27, 95% CI: 0.06-0.61), and ORR (0.27, 95% CI: 0.08-0.55) rates. Pembrolizumab- (0.06, 95% CI: 0.00-0.13) and toripalimab-based (0.02, 95% CI: 0.00-0.08) neoadjuvant immunochemotherapy had significantly lower incidence of 3 or higher grade TRAEs than other ICIs (p < 0.01).

## 4 Discussion

ICIs plus chemotherapy have emerged in the neoadjuvant therapy of NSCLC. This approach has demonstrated good therapeutic effects and safety, offering new hope for the prolonged survival of patients with NSCLC (86). It represents the current direction of NSCLC neoadjuvant therapy. However, there is still a need to further evaluate the efficacy, safety, and survival of this treatment for operable NSCLC. To address this, we conducted this meta-analysis. Our analysis, which included 60 studies and 3,632 patients, found that neoadjuvant immunochemotherapy was superior to neoadjuvant chemotherapy in terms of achieving higher rates of pCR, MPR, and ORR. Additionally, neoadjuvant immunochemotherapy was related to a lower incidence of surgical complications and longer 1-year and 2-year OS, without affecting TRAEs and R0 resection rates. These findings provide valuable reference for the clinical treatment of NSCLC.

Our study investigated the efficacy of neoadjuvant immunochemotherapy for NSCLC, and the results showed that the pooled pCR was 0.35 (95% CI: 0.31, 0.39), MPR was 0.59 (95% CI: 0.54, 0.64), and ORR was 0.71 (95% CI: 0.66, 0.76). These rates were significantly higher than those for neoadjuvant chemotherapy (pooled pCR of 0.04) and neoadjuvant immunotherapy (pCR of no more than 0.10) reported in previous studies (7, 15, 16). Combination therapy can achieve optimal treatment effects by stimulating tumor cell mutations, releasing new tumor antigens, and restructuring the immune microenvironment (87). Our study found that neoadjuvant immunochemotherapy performed better for patients with squamous cell carcinoma, or stage III (p < 0.01). Previous studies have also shown that neoadjuvant systemic therapy brings greater clinical benefits to patients with stage III, but caution is needed when assessing pathologic response due to bias introduced by non-operative patients (22). It is possible that patients with squamous cell carcinoma, or stage III are associated with a high level of tumor mutational burden (TMB), inflammation, and PD-L1 expression, which may make them more responsive to immunotherapy (88). However, it is crucial to remember that these factors are not absolute for individual patients.

In neoadjuvant immunochemotherapy for NSCLC, the pooled 1-year OS was 0.97 (95%CI: 0.96, 0.99), and 2-year OS was 0.89 (95%CI: 0.83, 0.94). The benefit of neoadjuvant chemotherapy of OS, compared to operation, is only 5%-6%. The CheckMate 816 trial showed that preoperative nivolumab in combination with chemotherapy resulted in a 37% lower risk of disease recurrence, progression, or death than chemotherapy (22). The SAKK 16/14 trial revealed an encouraging 1-year event-free survival (EFS) of 73% and 2-year EFS of 68% in the neoadjuvant durvalumab plus chemotherapy group (21). EFS measures the time from treatment initiation to the occurrence of any disease-related event and can provide an early assessment of treatment efficacy. However, we did not evaluate EFS in our study because the endpoint of survival is non-uniform, including EFS, OS, progression-free survival (PFS), and disease-free survival, making the survival outcomes difficult to analyze.

Our study suggested that neoadjuvant immunochemotherapy did not increase TRAEs compared with neoadjuvant chemotherapy and may lead to fewer surgical complications, fully confirming its safety. The pooled rate of all grade TRAEs was 0.60 (95% CI: 0.60, 0.81), and that of grade 3 or higher TRAEs was 0.24 (95% CI: 0.16, 0.32). In NSCLC, immune-related adverse events, including pneumonitis, thyroid dysfunction, and skin rash, are the most common types of TRAEs associated with ICIs used in neoadjuvant immunochemotherapy. The pooled rate of surgical complications of neoadjuvant immunochemotherapy was 0.13 (95% CI: 0.07, 0.18), and the pooled R0 resection rate of neoadjuvant immunochemotherapy was 0.98 (95% CI: 0.96, 0.99). Although these adverse events could be serious and potentially life-threatening, they are relatively rare and can usually be managed effectively if detected and treated early. Close monitoring and prompt reporting of any symptoms to the healthcare provider are essential for ensuring the safety of neoadjuvant immunochemotherapy in patients with NSCLC.

Accurately identifying the population for neoadjuvant immunotherapy is critical. Our data show that higher PD-L1 expression (TPS >= 50% or TPS = 1-49%) performed better in neoadjuvant immunochemotherapy (p < 0.01). In the published NADIM trial, pCR patients had a higher proportion of PD-L1 positive tumors, but PD-L1 expression was not related to patient survival (18). The results of the CheckMate 816 study revealed that patients with pretherapy PD-L1 above 1% had longer EFS than those with PD-L1 below 1%, supporting PD-L1 as a predictor of neoadjuvant immunotherapy (22). However, in the phase II study of atezolizumab plus chemotherapy, no significant difference was found in MPR and pretreatment PD-L1 (20). TMB is a measure of the number of mutations in a tumor’s DNA and has been suggested as a potential predictive biomarker for response to neoadjuvant immunochemotherapy (22). Additionally, the preoperative ctDNA clearance rate may be related to a high predictive effect on postoperative recurrence (22, 40). However, the mechanism and predictive value of ctDNA clearance still need further exploration in basic research. Although our data suggest that these biomarkers can be used as predictors, more marker guidance is needed for patient selection and precise treatment due to the heterogeneity of the data.

Moreover, no significant differences were observed in pCR (p = 0.92), MPR (p = 0.80), and ORR (p = 0.61) between 2 or more treatment cycles, suggesting that increasing cycles of therapy may not increase efficacy. Patients who were PR or CR were related to a higher MPR rate than those in SD (p < 0.01). We also found that pembrolizumab- or toripalimab-based neoadjuvant immunochemotherapy performed better in efficacy without affecting >= 3 grade TRAEs. In most included studies, neoadjuvant immunotherapy combined with chemotherapy was used for 2-4 cycles, and operation was performed 4–6 weeks after neoadjuvant immunotherapy (14).

Our study has some limitations. Firstly, the follow-up time of some trials was not long enough to adequately report on long-term survival. Additionally, existing studies are still limited regarding the selection of effective predictors such as ctDNA and the timing of neoadjuvant immunotherapy or adjuvant therapy, making it difficult to obtain more novel results. Thirdly, the study outcomes were non-uniform, making it difficult to pool the survival results of EFS. Therefore, more innovative long-term RCTs are needed to overcome the above obstacles, and the internal mechanism of neoadjuvant immunochemotherapy needs to be further explored. Despite these limitations, this meta-analysis provides objective information on the efficacy, safety, and survival of neoadjuvant immunochemotherapy in operable NSCLC.

## 5 Conclusion

Our study demonstrates the reliable efficacy, safety, and survival of neoadjuvant immunochemotherapy for operable NSCLC, making it a promising direction for neoadjuvant treatment in the future.

## Data availability statement

The original contributions presented in the study are included in the article/**Supplementary Material**. Further inquiries can be directed to the corresponding author.

## Author contributions

YZ: Conceptualization, Data curation, Formal analysis, Methodology, Software, Writing – original draft, Writing – review & editing. BF: Formal analysis, Methodology, Writing – original draft. JC: Formal analysis, Project administration, Writing – review & editing, Funding acquisition. LY: Conceptualization, Funding acquisition, Supervision, Investigation, Resources, Writing – review & editing.
