# Supplementary material for: Efficacy, safety, and survival of neoadjuvant immunochemotherapy in operable non-small cell lung cancer: a systematic review and meta-analysis
Source: Front Immunol. 2023 Dec 1;14:1273220. doi: 10.3389/fimmu.2023.1273220 (PMC10722296; doi:10.3389/fimmu.2023.1273220)
Supplement: Supplementary file 1 [file DataSheet_1.pdf]

Supplementary Material

Supplementary Figure 1 Comparison of secondary endpoints between neoadjuvant immunochemotherapy with neoadjuvant chemotherapy alone

Supplementary Figure 2 Pooled secondary endpoints of neoadjuvant immunochemotherapy in resectable non-small cell lung cancer

Supplementary Figure 3 Funnel plots of dual-arm studies

Supplementary Figure 4 Sensitivity analysis for primary outcomes of RCTs included

Supplementary Figure 5 Subgroup analysis of MPR by clinical characteristics

Supplementary Figure 6 Subgroup analysis of ORR by clinical characteristics

Supplementary Figure 7 Subgroup analysis of 3 or higher grade TRAEs by type of ICI

Supplementary Table 1 Search strategies

Supplementary Table 2 Quality assessment for RCTs though the Cochrane Collaboration’s Tool

Supplementary Table 3 Quality assessment for dual-arm non-randomized studies using the Newcastle-Ottawa Scale

Supplementary Table 4 Quality assessment for single-arm non-randomized studies using Methodological Index for Non-Randomized Studies Criteria

Supplementary Figure 1 Comparison of secondary endpoints between neoadjuvant immunochemotherapy with neoadjuvant chemotherapy alone

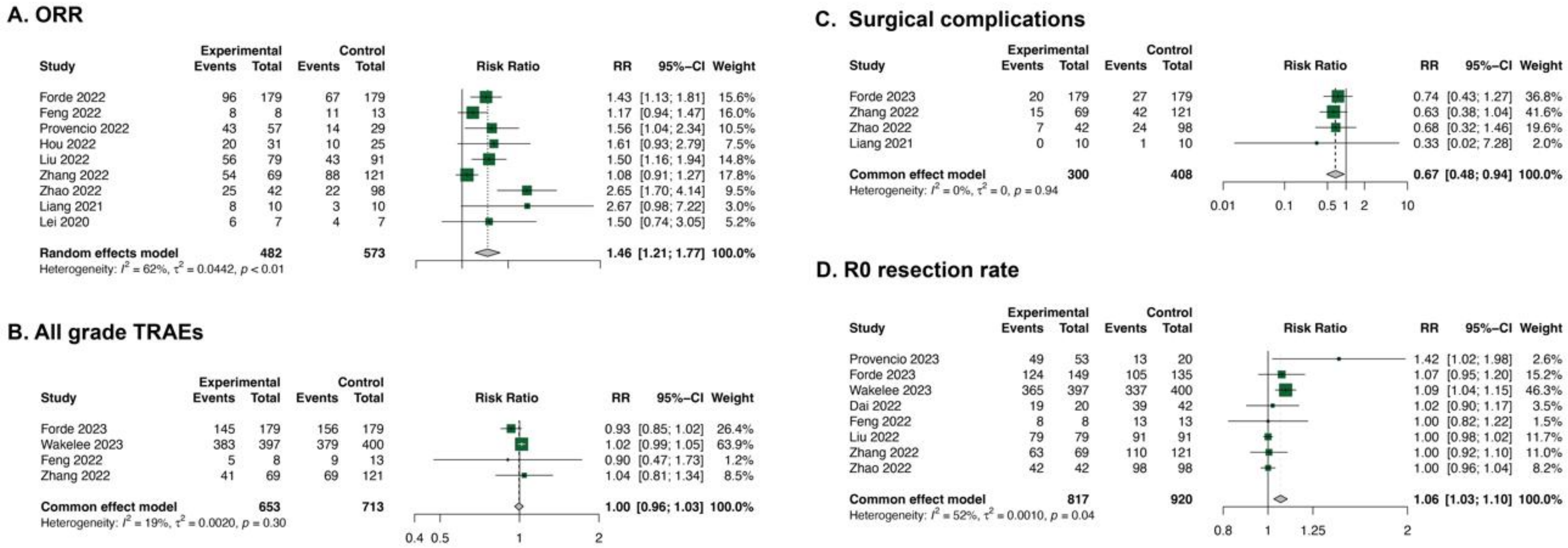

Supplementary Figure 2 Pooled secondary endpoints of neoadjuvant immunochemotherapy in resectable non-small cell lung cancer.

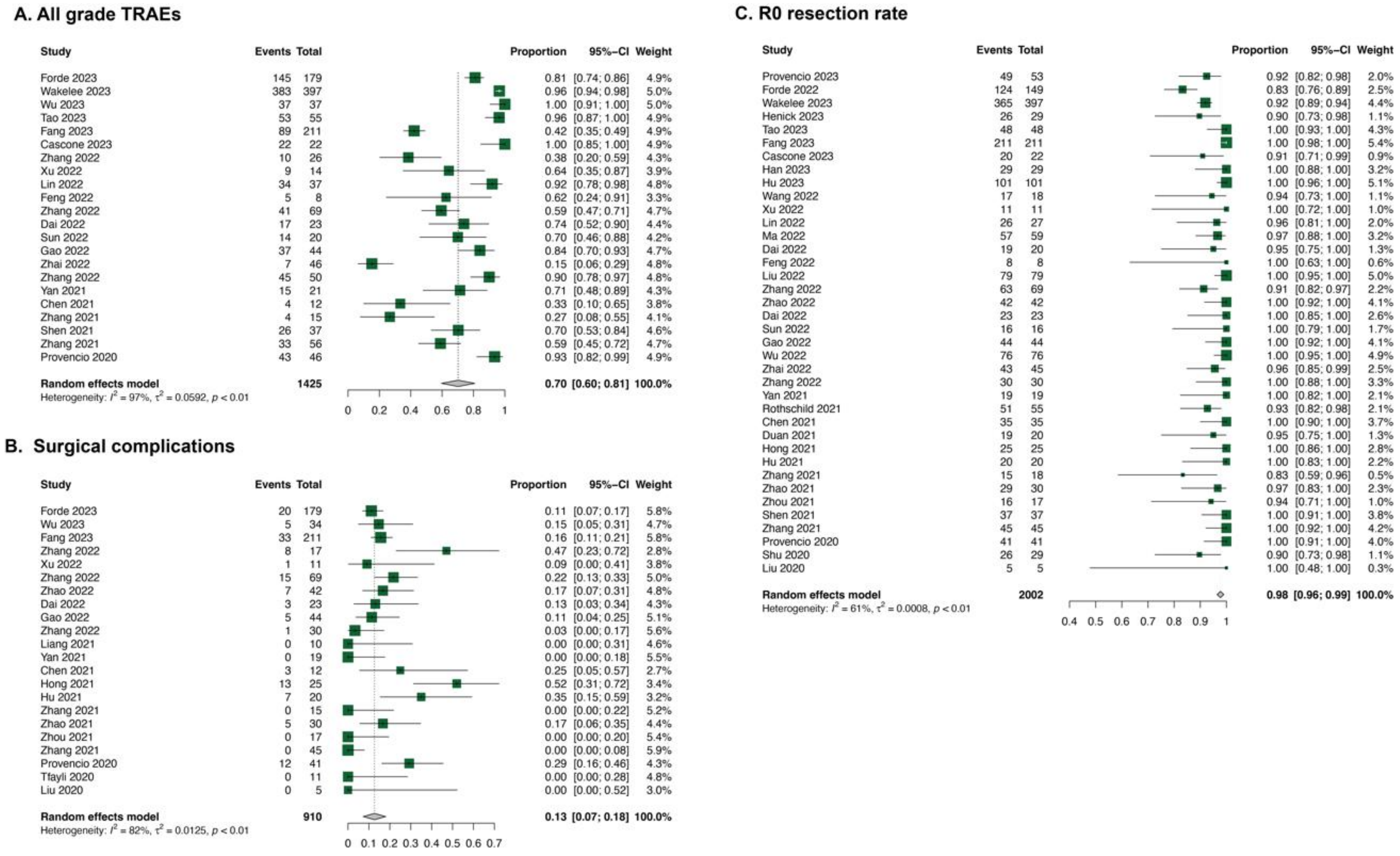

Supplementary Figure 3 Funnel plots of dual-arm studies

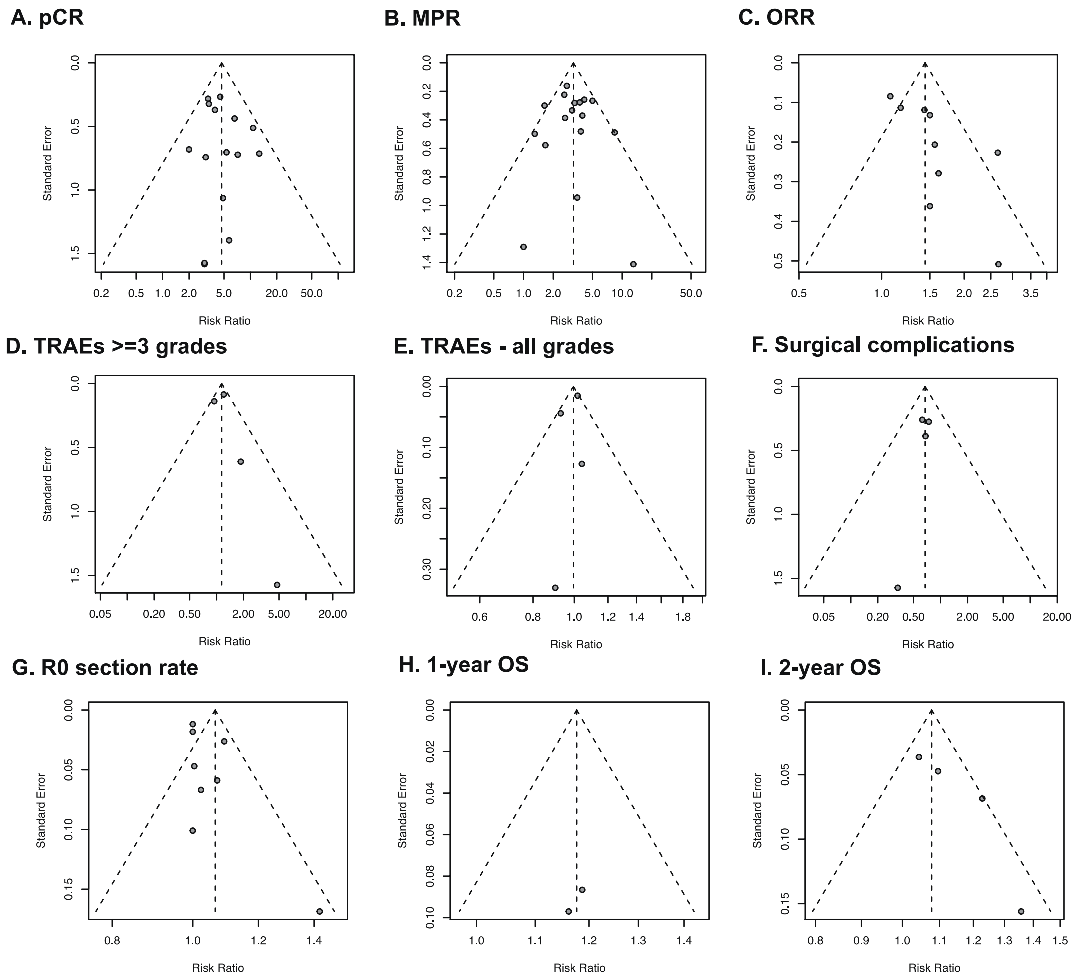

Supplementary Figure 4 Sensitivity analysis for primary outcomes of RCTs included

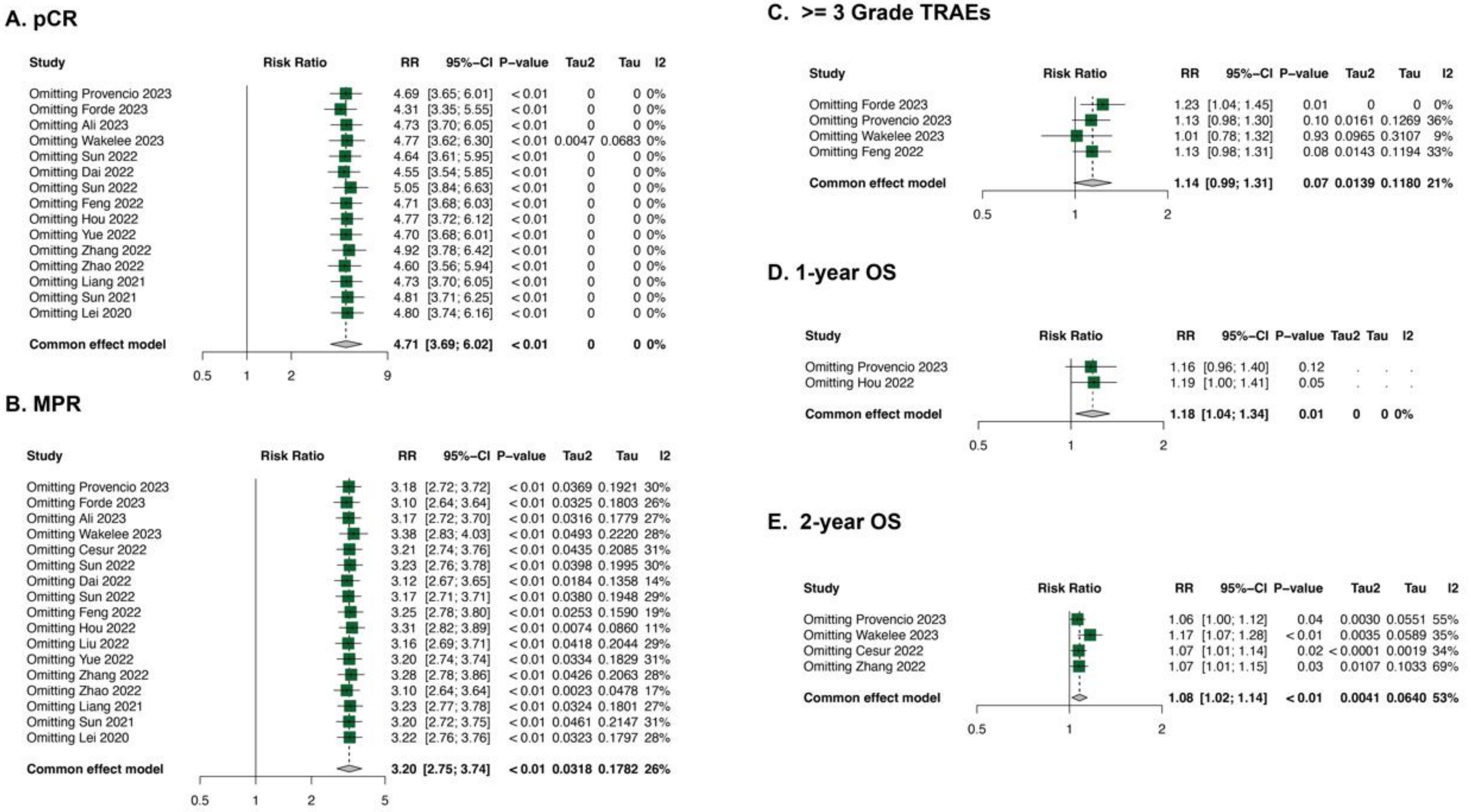

Supplementary Figure 5 Subgroup analysis of MPR by clinical characteristics

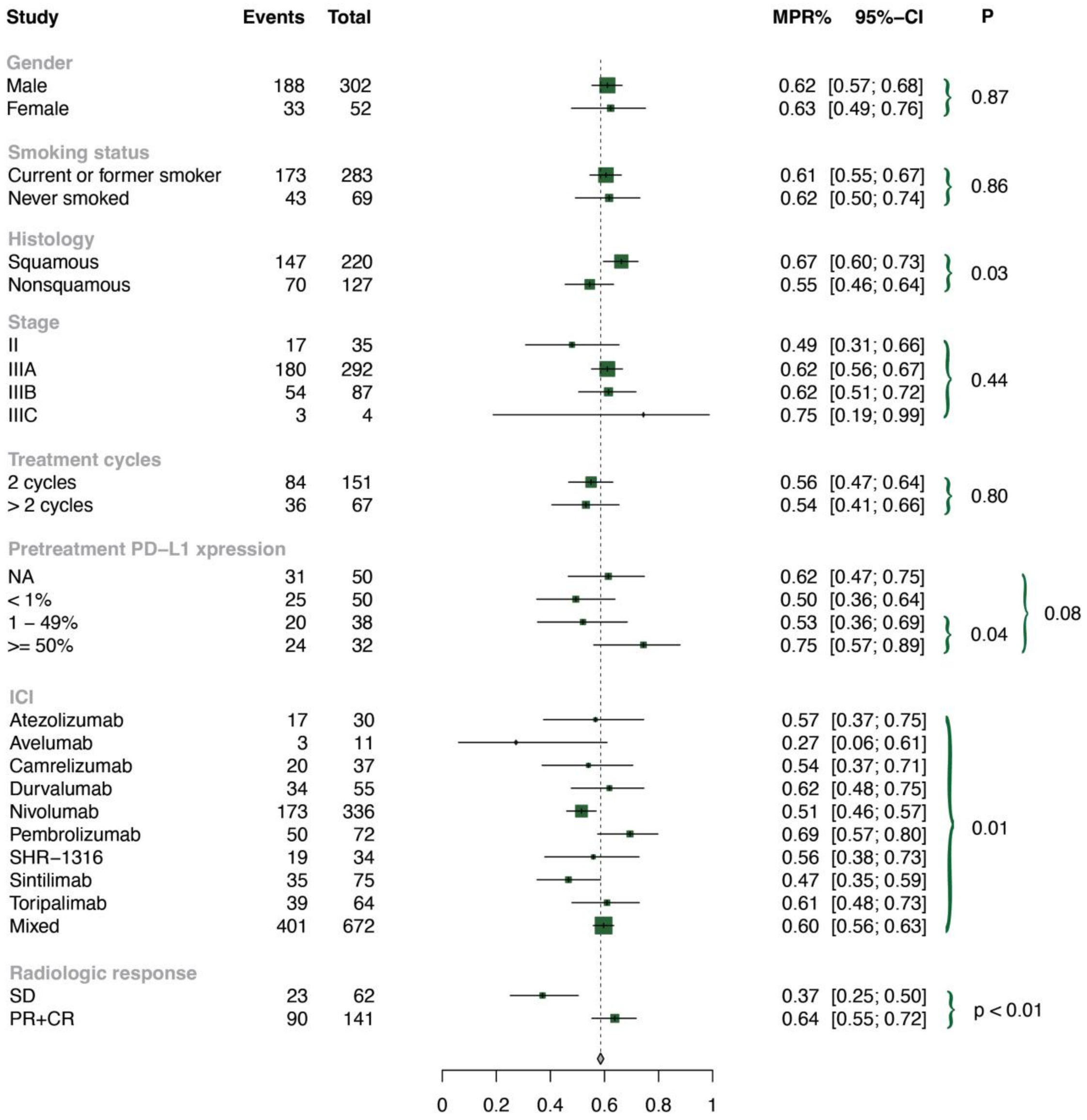

Supplementary Figure 6 Subgroup analysis of ORR by clinical characteristics

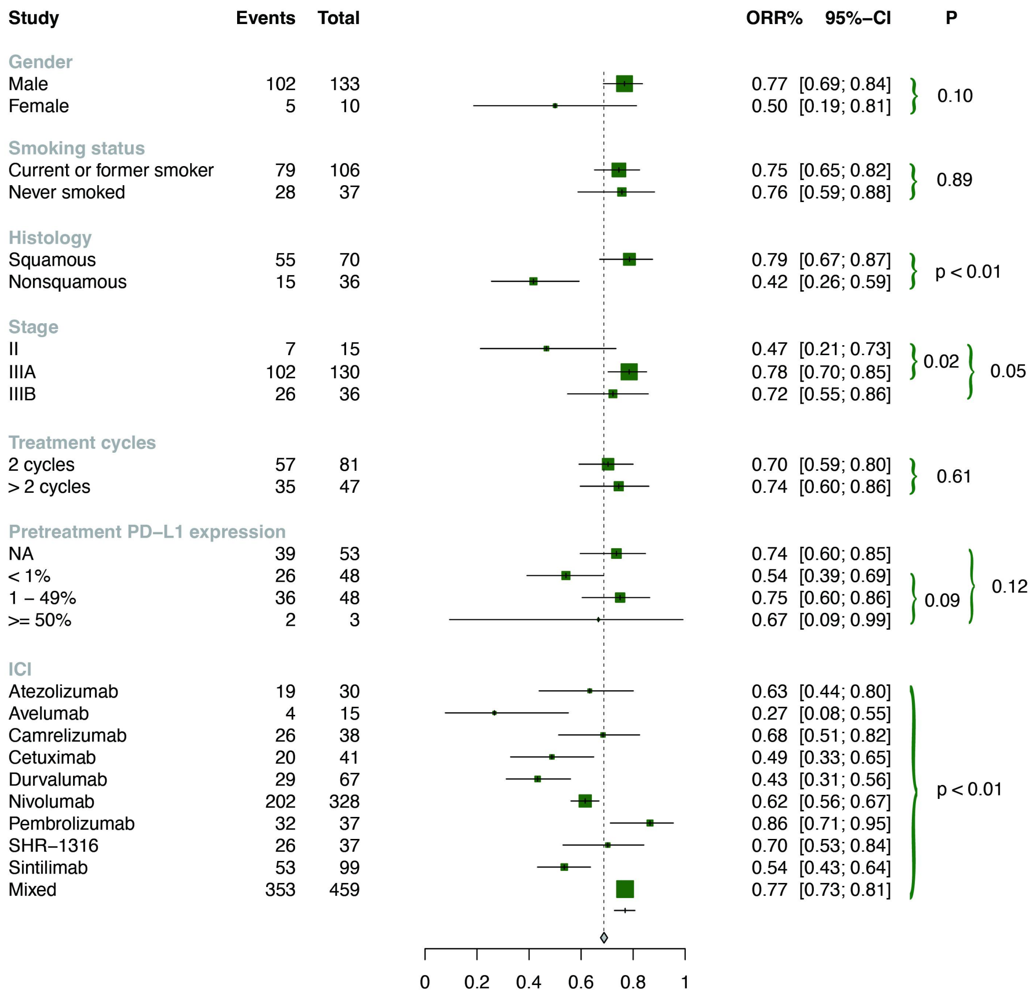

Supplementary Figure 7 Subgroup analysis of 3 or higher grade TRAEs by type of ICI

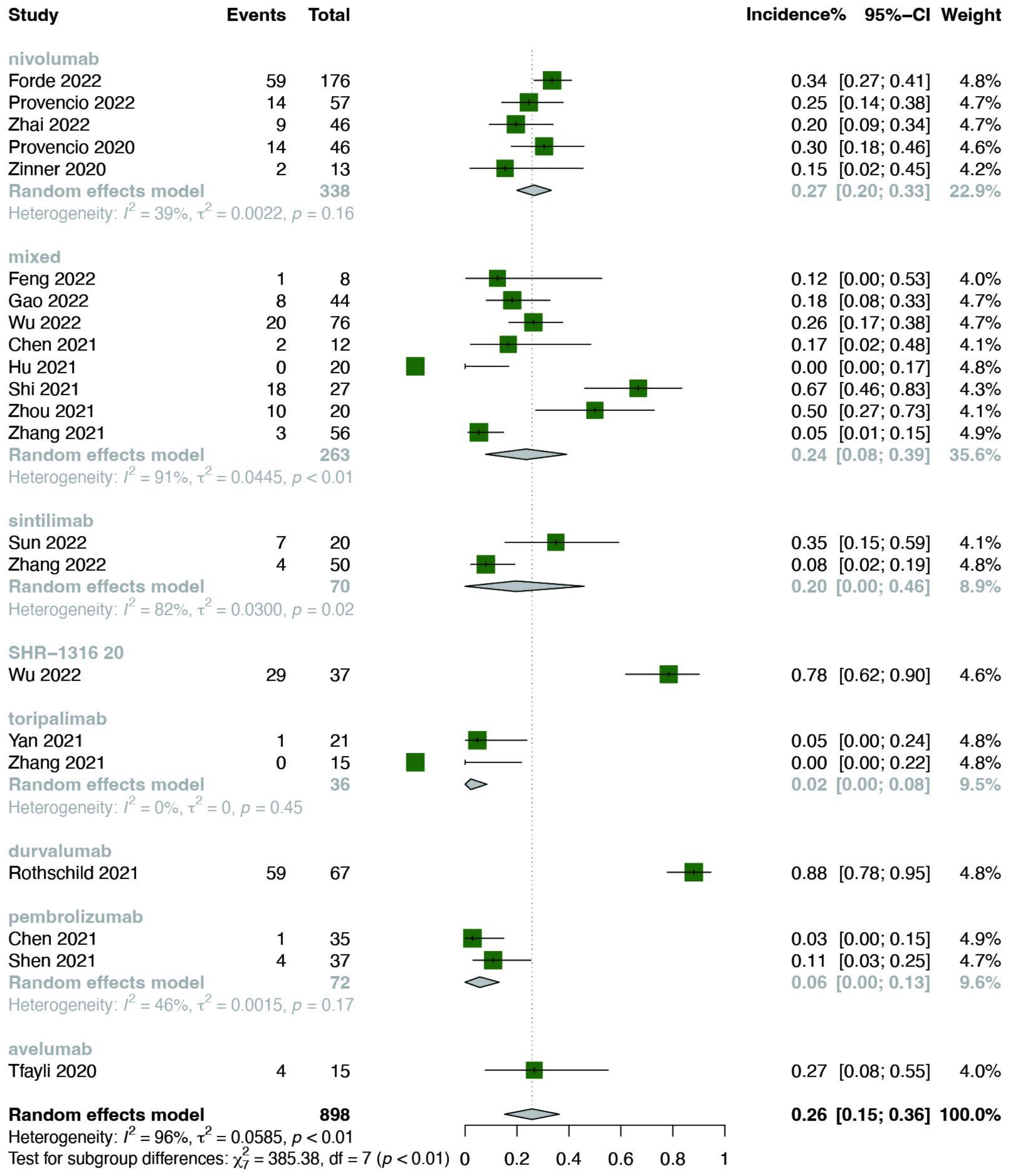

Supplementary Table 1 Search strategies

Database		Keywords
PubMed	#1	"Neoadjuvant Therapies"[All Fields] OR "neoadjuvant"[All Fields] OR "therapy neoadjuvant"[All Fields] OR "Neoadjuvant Treatment"[All Fields] OR "Neoadjuvant Treatments"[All Fields] OR "treatment neoadjuvant"[All Fields]
	#2	"immunotherapy"[All Fields] OR "immunotherapies"[All Fields] OR "immune checkpoint inhibitor"[All Fields] OR "PD-1"[All Fields] OR "PD-L1"[All Fields] OR "CTLA-4"[All Fields] OR "pembrolizumab"[All Fields] OR "atezolizumab"[All Fields] OR "nivolumab"[All Fields] OR "ipilimumab"[All Fields] OR "durvalumab"[All Fields] OR "tremelimumab"[All Fields] OR "camrelizumab"[All Fields] OR "tislelizumab"[All Fields] OR "sintilimab"[All Fields]
	#3	"therapy drug"[All Fields] OR "Drug Therapies"[All Fields] OR "therapies drug"[All Fields] OR "Chemotherapy"[All Fields] OR "Chemotherapies"[All Fields] OR "Pharmacotherapy"[All Fields] OR "Pharmacotherapies"[All Fields] OR "Cisplatin"[All Fields] OR "Carboplatin"[All Fields] OR "Gemcitabine"[All Fields] OR "Vinorelbine"[All Fields] OR "Docetaxel"[All Fields] OR "Pemetrexed"[All Fields] OR "Paclitaxel"[All Fields]
	#4	"carcinoma non small cell lung"[All Fields] OR "carcinomas non small cell lung"[All Fields] OR "lung carcinoma non small cell"[All Fields] OR "lung carcinomas non small cell"[All Fields] OR "Non-Small-Cell Lung Carcinomas"[All Fields] OR "non small cell lung carcinoma"[All Fields] OR "non-small-cell lung carcinoma"[All Fields] OR "non small cell lung carcinoma"[All Fields] OR "Nonsmall Cell Lung Cancer"[All Fields] OR "carcinoma non small cell lung"[All Fields] OR "Non-Small Cell Lung Cancer"[All Fields] OR "NSCLC"[All Fields]
	#5	#1 AND #2 AND #3 AND #4
Embase	#1	'neoadjuvant therapies' OR 'therapy, neoadjuvant' OR 'neoadjuvant treatment' OR 'neoadjuvant treatment' OR 'neoadjuvant treatments' OR 'treatment, neoadjuvant' OR 'neoadjuvant'
	#2	'immunotherapy' OR 'immunotherapies' OR 'immune checkpoint inhibitor' OR 'pd-1' OR 'pd-l1' OR 'ctla-4' OR 'pembrolizumab' OR 'atezolizumab' OR 'nivolumab' OR 'ipilimumab' OR 'durvalumab' OR 'tremelimumab' OR 'camrelizumab' OR 'tislelizumab' OR 'sintilimab'
	#3	'therapy, drug' OR 'drug therapies' OR 'therapies, drug' OR 'chemotherapy' OR 'chemotherapies' OR 'pharmacotherapy' OR 'pharmacotherapies' OR 'cisplatin' OR 'carboplatin' OR 'gemcitabine' OR 'vinorelbine' OR 'docetaxel' OR 'pemetrexed' OR 'paclitaxel'
	#4	carcinoma, non small cell lung' OR 'carcinomas, non-small-cell lung' OR 'lung carcinoma, non-small-cell' OR 'lung carcinomas, non-small-cell' OR 'non-small-cell lung carcinomas' OR 'non-small cell lung carcinoma' OR 'non-small-cell lung carcinoma' OR 'non small cell lung carcinoma' OR 'nonsmall cell lung cancer' OR 'carcinoma, non-small cell lung' OR 'non-small cell lung cancer' OR 'NSCLC'
	#5	#1 AND #2 AND #3 AND #4
Wed of Science	#1	“Neoadjuvant Therapies” OR “Therapy, Neoadjuvant” OR “Neoadjuvant Treatment” OR “Neoadjuvant Treatments” OR “Treatment, Neoadjuvant” OR “Neoadjuvant”
	#2	“immunotherapy” OR “immunotherapies” OR “immune checkpoint inhibitor” OR “PD-1” OR “PD-L1” OR “CTLA-4” OR “pembrolizumab” OR “atezolizumab” OR “nivolumab” OR “ipilimumab” OR “durvalumab” OR “tremelimumab” OR “camrelizumab” OR “tislelizumab” OR “sintilimab”

	#3	“Therapy, Drug” OR “Drug Therapies” OR “Therapies, Drug” OR “Chemotherapy” OR “Chemotherapies” OR “Pharmacotherapy” OR “Pharmacotherapies” OR “Cisplatin” OR “Carboplatin” OR “Gemcitabine” OR “Vinorelbine” OR “Docetaxel” OR “Pemetrexed” OR “Paclitaxel”
	#4	“Carcinoma, Non Small Cell Lung” OR “Carcinomas, Non-Small-Cell Lung” OR “Lung Carcinoma, Non-Small-Cell” OR “Lung Carcinomas, Non-Small-Cell” OR “Non-Small-Cell Lung Carcinomas” OR “Non-Small Cell Lung Carcinoma” OR “Non-Small-Cell Lung Carcinoma” OR “Non Small Cell Lung Carcinoma” OR “Nonsmall Cell Lung Cancer” OR “Carcinoma, Non-Small Cell Lung” OR “Non-Small Cell Lung Cancer” OR “NSCLC”
	#5	#1 AND #2 AND #3 AND #4
**Cochrane Library**	#1	"neoadjuvancy" OR "neoadjuvant therapy" OR "neoadjuvant therapy" OR "neoadjuvant" OR "neoadjuvants" OR "neoadjuvent" in All Text
	#2	"immunotherapy" OR "immunotherapies" in All Text
	#3	"drug therapy" OR "chemotherapies" OR "chemotherapy" in All Text
	#4	"carcinoma, non-small-cell lung" OR "non-small-cell lung carcinoma" OR "non small cell lung cancer" in All Text
	#5	#1 AND #2 AND #3 AND #4 - (Word variations have been searched)

Supplementary Table 2 Quality assessment for RCTs though the Cochrane Collaboration's Tool

**Author (Year)**	**Randomization**	**Allocation concealment**	**Blinding of participants and personnel**	**Blinding of outcome assessment**	**Incomplete outcome data**	**Selective reporting**	**Other bias**
Wakelee (2023)	Low risk	Low risk	Low risk	Low risk	Low risk	Low risk	Low risk
Provencio (2023)	Low risk	Low risk	Low risk	Low risk	Low risk	Low risk	Low risk
Forde (2023)	Low risk	Low risk	Low risk	Low risk	Low risk	Low risk	Low risk
Lei (2020)	Low risk	Low risk	Low risk	Low risk	Low risk	Low risk	Low risk

Supplementary Table 3 Quality assessment for dual-arm non-randomized studies using the Newcastle-Ottawa Scale

Author (Year)	Selection				Comparability	Outcome			Quality score
	Representativeness of exposed cohort	*Comparability of cohorts*	*Ascertainment of exposure*	*Outcome present at start of study*	*Comparability of cohorts*	*Assessment of Outcome*	*Length of follow-up*	*Adequacy of follow-up*	
Alì (2023)	★	★	★	★	★	★	★	★	8
Cesur (2022)	★	★	★	★	★	★	★	★	8
Sun (2022)	★	★	★	★	★★	★	★	★	9
Dai (2022)	★	★	★	★	★★	★	★	★	9
Sun (2022)	★	★	★	★	★	★	★	★	8
Feng (2022)	★	★	★	★	★	★	★	★	8
Hou (2022)	★	★	★	★	★★	★	★	★	9
Liu (2022)	★	★	★	★	★★	★	★	★	9
Yue (2022)	★	★	★	★		★	★	★	7
Zhang (2022)	★	★	★	★	★	★	★	★	8
Zhao (2022)	★	★	★	★		★		★	6
Liang (2021)	★	★	★	★	★★	★	★	★	9
Sun (2021)	★	★	★	★	★★	★	★	★	9

Supplementary Table 4 Quality assessment for single-arm non-randomized studies using Methodological Index for Non-Randomized Studies Criteria

**Criteria for assessment**	**Clearly stated aim**	**Inclusion of consecutive patients**	**Prospective data collection**	**Endpoints appropriate of study**	**Unbiased assessment of study endpoint**	**Follow-up period appropriate**	**Loss to follow up less than 5%**	**Prospective calculation of the study size**	**Adequate control group**	**Contemporary groups**	**Baseline equivalence of groups**	**Adequate statistical analysis**	**Total score**
Henick (2023)	2	2	2	2	0	2	2	0	*	*	*	*	12
Tao (2023)	2	2	2	2	0	1	2	0	*	*	*	*	11
Fang (2023)	2	2	2	2	0	2	2	0	*	*	*	*	12
Cascone (2023)	2	2	2	2	0	2	2	0	*	*	*	*	12
Chen (2023)	2	2	2	2	0	1	2	0	*	*	*	*	11
Zhao (2023)	2	2	2	2	0	1	2	0	*	*	*	*	11
Han (2023)	2	2	2	2	0	1	2	0	*	*	*	*	11
Hu (2023)	2	2	2	2	0	2	2	0	*	*	*	*	12
Zhuang (2023)	2	2	2	2	0	1	2	0	*	*	*	*	11
Wu (2023)	2	2	2	2	2	2	2	0	*	*	*	*	14
Wang (2022)	2	2	2	2	0	1	2	0	*	*	*	*	11
Zhang (2022)	2	2	2	2	0	1	2	0	*	*	*	*	11
Xu (2022)	2	2	2	2	0	1	2	0	*	*	*	*	11
Lin (2022)	2	2	2	2	0	1	2	0	*	*	*	*	11
Dong (2022)	2	2	2	2	0	2	2	0	*	*	*	*	12

Ma (2022)	2	2	2	2	0	1	2	0	*	*	*	*	11
Dai (2022)	2	2	2	2	0	2	2	0	*	*	*	*	12
Faehling (2022)	2	2	2	2	0	2	2	0	*	*	*	*	12
Sun (2022)	2	2	2	2	0	1	2	0	*	*	*	*	11
Gao (2022)	2	2	2	2	0	2	2	0	*	*	*	*	12
Qiu (2022)	2	2	2	2	0	2	2	0	*	*	*	*	12
Wu (2022)	2	2	2	2	0	1	2	0	*	*	*	*	11
Zhai (2022)	2	2	2	2	0	2	2	0	*	*	*	*	12
Zhang (2022)	2	2	2	2	0	2	2	0	*	*	*	*	12
Yan (2021)	2	2	2	2	0	2	2	0	*	*	*	*	12
Rothschild (2021)	2	2	2	2	0	2	2	0	*	*	*	*	12
Chen (2021)	2	2	2	2	0	2	2	0	*	*	*	*	12
Chen (2021)	2	2	2	2	0	2	2	0	*	*	*	*	12
Duan (2021)	2	2	2	2	0	2	2	0	*	*	*	*	12
Hong (2021)	2	2	2	2	0	2	2	0	*	*	*	*	12
Hu (2021)	2	2	2	2	0	2	2	0	*	*	*	*	12
Shi (2021)	2	2	2	2	0	2	2	0	*	*	*	*	12
Zhang (2021)	2	2	2	2	0	2	2	0	*	*	*	*	12
Zhao (2021)	2	2	2	2	0	2	2	0	*	*	*	*	12
Zhou (2021)	2	2	2	2	0	2	2	0	*	*	*	*	12
Shen (2021)	2	2	2	2	0	2	2	0	*	*	*	*	12

Zhang (2021)	2	2	2	2	0	2	2	0	*	*	*	*	12
Cascone (2020)	2	2	1	2	0	2	2	0	*	*	*	*	12
Provencio (2020)	2	2	2	2	0	2	2	0	*	*	*	*	12
Shu (2020)	2	2	2	2	0	2	2	0	*	*	*	*	12
Tfayli (2020)	2	2	2	2	0	2	2	0	*	*	*	*	12
Zinner (2020)	2	2	2	2	0	2	2	0	*	*	*	*	12
Liu (2020)	2	2	2	2	0	2	2	0	*	*	*	*	12
Hilbe (2015)	2	2	2	2	0	2	2	0	*	*	*	*	12
